# Latent Myofascial Trigger Points Injection Reduced the Severity of Persistent, Moderate to Severe Allergic Rhinitis: A Randomized Controlled Trial

**DOI:** 10.3389/fmed.2021.731254

**Published:** 2021-10-01

**Authors:** Yu Liu, Yan Yang, Qiya Hu, Ahmed Badughaish, Hanbing Zhang, Feng Qi, Yuedong Hou

**Affiliations:** ^1^Department of Anesthesiology and Pain Clinic, Qilu Hospital, Cheeloo College of Medicine, Shandong University, Ji'nan, China; ^2^Department of Otolaryngology, Qilu Hospital, Cheeloo College of Medicine, Shandong University, Ji'nan, China; ^3^Department of Anesthesiology, Qilu Hospital, Cheeloo College of Medicine, Shandong University, Ji'nan, China

**Keywords:** allergic rhinitis, latent myofascial trigger points, autonomic nerve network, sublingual immunotherapy, treatment

## Abstract

**Background:** Myofascial trigger points (MTrPs) injection has been effectively used for the management of chronic painful diseases. Latent MTrPs can induce autonomic nerve phenomena. In our clinic, we observed that allergic rhinitis (AR) symptoms significantly improved when latent MTrPs injection was performed for migraine.

**Objective:** To compare the efficacy and safety between latent MTrPs injection and sublingual immunotherapy (SLIT) in patients with persistent, moderate to severe AR.

**Methods:** This randomized controlled trial was conducted with 112 patients with AR. Patients were randomized to receive SLIT (*n* = 56) or latent MTrPs injection. Total nasal symptom score (TNSS, *n* = 56), nasal symptoms, medication days, and adverse events were evaluated during the 9 months follow-up period after treatment in both groups.

**Results:** Latent MTrPs injection significantly reduced TNSS to a greater level from baseline (from 8.36 ± 1.96 to 4.43 ± 2.18) than SLIT (from 8.66 ± 2.31 to 7.80 ± 2.47) at week 1 (*P* < 0.001), and sustained the improvement in symptoms throughout to month 9. Latent MTrPs showed statistically significant differences *vs*. SLIT for the TNSS reduction both at month 2 (6.59 ± 2.37 vs. 2.64 ± 2.38; *p* < 0.001) and month 3 (4.59 ± 2.77 vs. 2.62 ± 2.43; p <0.001). Latent MTrPs also showed a better improvement in the onset time of efficacy compared with SLIT. Adverse reactions were few and non-serious in both treatment groups.

**Conclusions:** Latent MTrPs injection significantly improved symptoms and decreased symptom-relieving medication use in patients with AR and was well tolerated.

**Clinical Trials Registration:** Chinese Clinical Trial Registry, ChiCTR1900020590. Registered 9 January 2019, http://www.chictr.org.cn/index.aspx.

## Introduction

Allergic rhinitis (AR) is a worldwide health problem and the prevalence of AR in the global population is approximately 10 to 20 % on the basis of physician diagnosis and as high as 20 % on the basis of self-reported nasal or eye symptoms (1, 2). AR not only affects the quality of sleep and work performance but also causes an estimated loss of 2 to 4 billion dollars in annual productivity per year (3). Despite the availability of management guidelines, persistently uncontrolled AR is common (4, 5).

Myofascial pain syndrome (MPS) is a clinical syndrome characterized by pain *via* palpation that is identified by myofascial trigger points (MTrPs). The lifetime prevalence of MPS is estimated to be 85 % (6). MTrPs can be divided into active and latent MTrPs according to the presence of spontaneous pain. Active MTrPs refer to points in skeletal muscles that produce spontaneous pain or pain in response to movement, while points that produce pain or discomfort when compressed are referred to as latent MTrPs (7). Latent MTrPs can induce non-pain-related symptoms such as changes in skin temperature, sweating, tearing, and other autonomous nerve responses (8). A recent study reported the occurrence of non-painful disease that is associated with latent MTrPs (9).

In recent years, the efficacy and safety of sublingual immunotherapy (SLIT) have been confirmed in some countries and regions, and SLIT has been widely used in clinical practice. However, SLIT needs to be used continuously for a long time and usually takes several months to induce an effect (10, 11). In our clinic, many patients with AR reported that their AR symptoms improved after latent MTrPs injection for migraine. We considered that was not a rare phenomenon. Active MTrPs can cause pain, whereas latent MTrPs can bring about autonomic phenomena (8). Several lines of evidence have emphasized the importance of innervation and AR symptoms (12, 13). Consequently, we hypothesized that latent MTrPs may be associated with AR. We proposed that latent MTrPs affect the autonomic nervous system and abnormal secretion of mucous from the nasopharyngeal glands.

Based on the above hypothesis, we conducted an open-label, randomized, controlled trial to examine the efficacy and safety of latent MTrPs injection in patients with persistent, moderate to severe AR. In the current study, we show that administration of latent MTrPs alleviates AR symptoms for several months and provide clinical evidence of the relationship between latent MTrPs and AR.

## Materials and Methods

### Trial Design

An open-label randomized control trial was conducted at Qilu Hospital, Cheeloo College of Medicine of Shandong University, Jinan, China. This study was approved by the Human Research Ethics Committee of Qilu Hospital (KYLL-2017-611) and registered with the Clinical Trial Registry Center (ChiCTR1900020590).

This trial included a run-in period of 14 days (baseline), a treatment process, and an observational period. In this study, a total of 112 patients were randomly divided into the following two groups: SLIT and latent MTrPs groups. An assistant performed randomization by generating random numbers in EXCEL (Microsoft. Corp) software. Patients assigned to the SLIT group took SLIT drops according to the instructions. Patients assigned to latent MTrPs group performed treatment procedure by the same practitioner. After treatment, the patients were followed for 9 months. All subjects confirmed informed consent regarding their participation in the study. All minor patients (<18 years of age) confirmed this study and their legal representatives confirmed informed consent. Patients were recruited from the department of otolaryngology of Qilu Hospital of Shandong University.

### Participants

Patients who were diagnosed with moderate to severe persistent AR to house dust mites were included. The diagnosis of dust mite AR was made by an otolaryngologist based on medical history, symptoms, signs, serum-specific immunoglobulin E (IgE), and positive allergen skin prick test (SPT). Persistent AR was defined as symptoms for 4 days or more per week for 4 or more consecutive weeks, and resulting in substantial impacts on daily life and work performance (3, 14). The other patient inclusion criteria were as follows: (1) any sex or ethnicity; (2) the use of contraception if female; and (3) no drug use (including glucocorticoids and antihistamines) to alleviate the symptoms of AR within 2 weeks prior to study entry. The exclusion criteria were as follows: (1) pregnancy or breastfeeding; (2) nasal polyps, sinusitis, or an obvious deviated nasal septum; (3) the long-term use of corticosteroids or immunosuppressive agents; and (4) cerebral vascular, lung, liver, kidney, or severe cardiovascular diseases.

### Intervention

#### Latent MTrPs Injection Procedures

Medications required for latent MTrPs injection included 5 ml of 2 % lidocaine (ZhaoHui Corp, Shanghai City, China), compound betamethasone injection (MSD Merck Sharp & Dohme AG, Switzerland), and 1,000 μg of vitamin B_12_ (JinYao Corp, Tianjin City, China) (8, 15). These medicines were diluted to 20 ml with 0.9 % saline. Latent MTrPs injection was performed using a 25 gauge needle (0.5^*^36 mm) and a 20 ml syringe (WeGo Corp, Weihai City, China). All palpation and injection procedures were performed by the same practitioner who had 20 years of experience. All participants in the latent MTrPs group received latent MTrPs injection treatment only once.

Before injection, it was essential to locate the latent MTrPs by palpation. Interestingly, latent MTrPs were always found in medial pterygoid muscles, lateral pterygoid muscles, sternocleidomastoid muscles, semispinalis, and splenius capitis muscles on each patient with AR by palpation. The sign of accurate latent MTrP insertion is pain of patient (referred pain and patient pain recognition) and/or muscle local twitch responses (LTRs) (8).

#### Lateral Pterygoid Muscles

**Figure 1:**
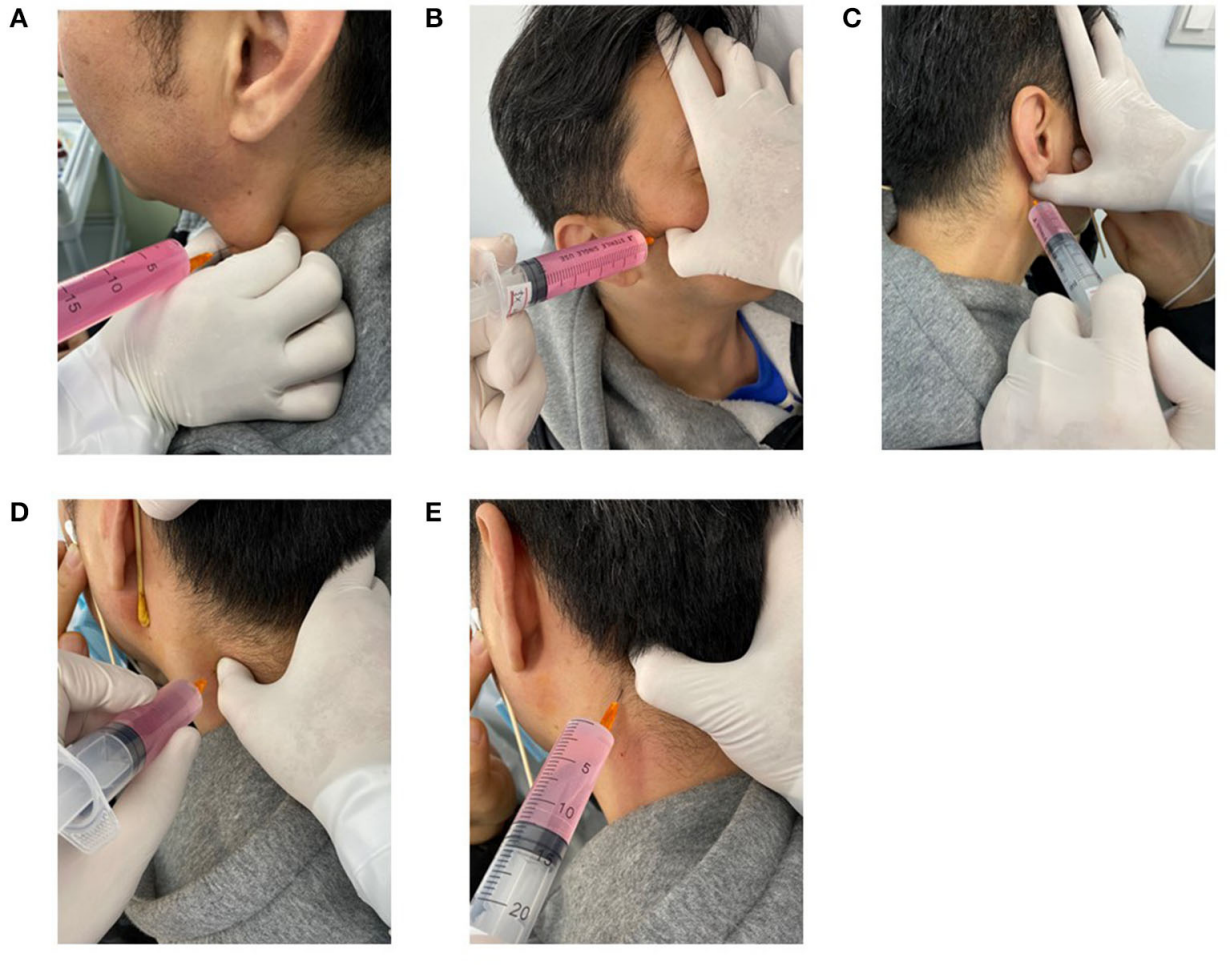
Performance of the latent MTrPs injection for AR. Sternocleidomastoid muscles latent MTrPs injection **(A)**. Lateral pterygoid muscles latent MTrPs injection **(B)**. Medial pterygoid muscles latent MTrPs injection **(C)**. Splenius capitis and Semispinalis muscles latent MTrPs injection **(D, E)**. MTrPs, myofascial trigger points.

Patients were seated on a chair with their shoulders and head against the wall to increase stability during palpation and injection (Figure 1).

Latent MTrPs palpation and injection procedures were performed in compliance with the technique described by Simons et al. (8). Deep finger pressure was applied on the surface of the skin with the thumb using systematic palpation as explained in the Simons manual to identify the latent MTrPs in the lateral pterygoid muscles, and the skin was sterilized with an appropriate antiseptic solution. Once the latent MTrPs were detected, the thumb of one hand remained in a fixed position on the skin, the patient was asked to keep his/her mouth open, and the other hand was used to hold the syringe and insert it into the muscles to identify the exact location by eliciting referred pain. When the patient felt referred pain, the phenomenon indicated that the needle was inserted into the latent MTrPs or approached the latent MTrPs, and then approximately 3 ml of liquid drugs was injected. The syringe was withdrawn before each injection to avoid intravascular injection. The other lateral muscles were injected using the same technique.

#### Medial Pterygoid Muscles

The medial pterygoid muscles were bilaterally injected using the same technique. This area is rich in blood vessels, so injection was performed carefully to avoid injuring the blood vessels.

#### Sternocleidomastoid Muscles

The sternocleidomastoid muscles were bilaterally injected by the same technique. When palpating the sternocleidomastoid muscle, the head was bent slightly laterally to relax the muscle, and the sternocleidomastoid muscle was pinched with the thumb and index finger to identify the latent MTrPs.

#### Semispinalis and Splenius Capitis Muscles

The patient sat with his/her arms crossed on the table and his/her forehead on his/her arm. The semispinalis and splenius capitis muscles were identified and palpated to locate latent MTrPs. After the injection was completed, the syringe was removed, and gentle pressure was applied to the injection site.

#### Sublingual Immunotherapy

*Dermatophagoides farinae* drops, which are produced by the Wolwo Biopharmaceutical Corp. Ltd. (Zhejiang, China), were used for SLIT. According to the instructions of the manufacturer, the drops were labeled with different concentrations of total protein (Bottle No. 1, 1 μg /ml; No. 2, 10 μg /ml; No. 3, 100 μg /ml; No. 4, 333 μg /ml; No. 5, 1,000 μg /ml). No. 1 was administrated with increasing doses, respectively, as 1, 2, 3, 4, 6, 8, and 10 drops (each drops 50 μL) day after day in the first week. No. 2 and No. 3 were administrated in the same way. After 3 weeks, No. 4 was administrated three drops per day in the fourth and fifth week. No. 5 was administrated two drops per day as maintenance therapy from the sixth week. SLIT was performed with *Dermatophagoides farinae* drops according to the schedules recommended by the manufacturer. The procedure was performed in strict accordance with the instructions under the guidance of an otolaryngologist.

### Primary and Secondary Endpoint

The primary endpoint was the TNSS at month 3, the secondary endpoints were each symptom score and medications days during the follow-up period (16). The scores were obtained before treatment (baseline) and 1 week, 2 weeks, 1 month, 2 months, 3 months, 6 months, and 9 months after treatment. The period from the beginning of treatment to the end of follow-up was 9 months.

The TNSS considers four common symptoms of AR: rhinorrhea, nasal obstruction, sneezing, and nasal itching. Each symptom is scored from 0 to 3 according to severity (11, 17). The sum of the scores for all symptoms was calculated and that score was considered the primary endpoint. The following scoring system was used. The absence of symptoms was scored as 0. The presence of obvious symptoms with rare discomfort was scored as (1). The presence of obvious but tolerable discomfort was scored as (2). Symptoms that were severe enough to be unbearable and that affected the daily life and sleep of the patient were scored as (3). The range of the primary endpoint was 0 to 12.

In the case of allergic symptoms, the participants had free access to rescue medication depending on the persistence and severity of symptoms. Rescue medication was fluticasone propionate nasal spray (Glaxo Wellcome, S.A. Spain: 50 ug fluticasone propionate per spray, two sprays per nostril every 12 hours, maximum usage 200 μg/d as needed). Participants were allowed to use rescue medication if their AR symptoms became intolerable. All participants were required to record the number of days of rescue medication usage, as the second endpoint, during the past week prior to the assessment points.

### Safety

Data on adverse events were collected and assessed after initial treatment and during the whole follow-up period. The adverse events of SLIT were completed daily by each patient (or by guardians) to document any local or systemic side effects after treatment. After completing latent MTrPs injection, the observations were made for 30 minutes before the patient left the clinic. In addition, the use of rescue medication that caused side effects were recorded, as well as vital signs, cardio-cerebrovascular accident, and significant changes of weight status were recorded.

### Statistical Analysis

We calculated that 31 participants per group would provide the trial with at least 95 % power (2-sided α = 0.05) to detect differences in the primary endpoint (TNSS) between latent MTrPs injection and SLIT, assuming a 20% dropout rate. Efficacy analyses were performed in the modified intention-to-treat population, which included all patients who underwent randomization, took an initial treatment of latent MTrPs and SLIT, recorded a baseline for the severity of the AR, and recorded the severity of AR after initial treatment or recorded use of medication prior to 1 week at each assessment point. Safety analyses were conducted in all patients who underwent randomization and who performed latent MTrPs or SLIT. Statistical analysis was performed using statistical product and service solutions 23.0 statistical software and GraphPad Prism 8.2.1 software. Measurement data are described as the means and SDs. Statistical analysis was performed using unpaired *t*-tests, nonparametric tests (Mann-Whitney tests), and chi-square tests. *P* < 0.05 was considered statistically significant.

## Results

### Study Patients

**Figure 2:**
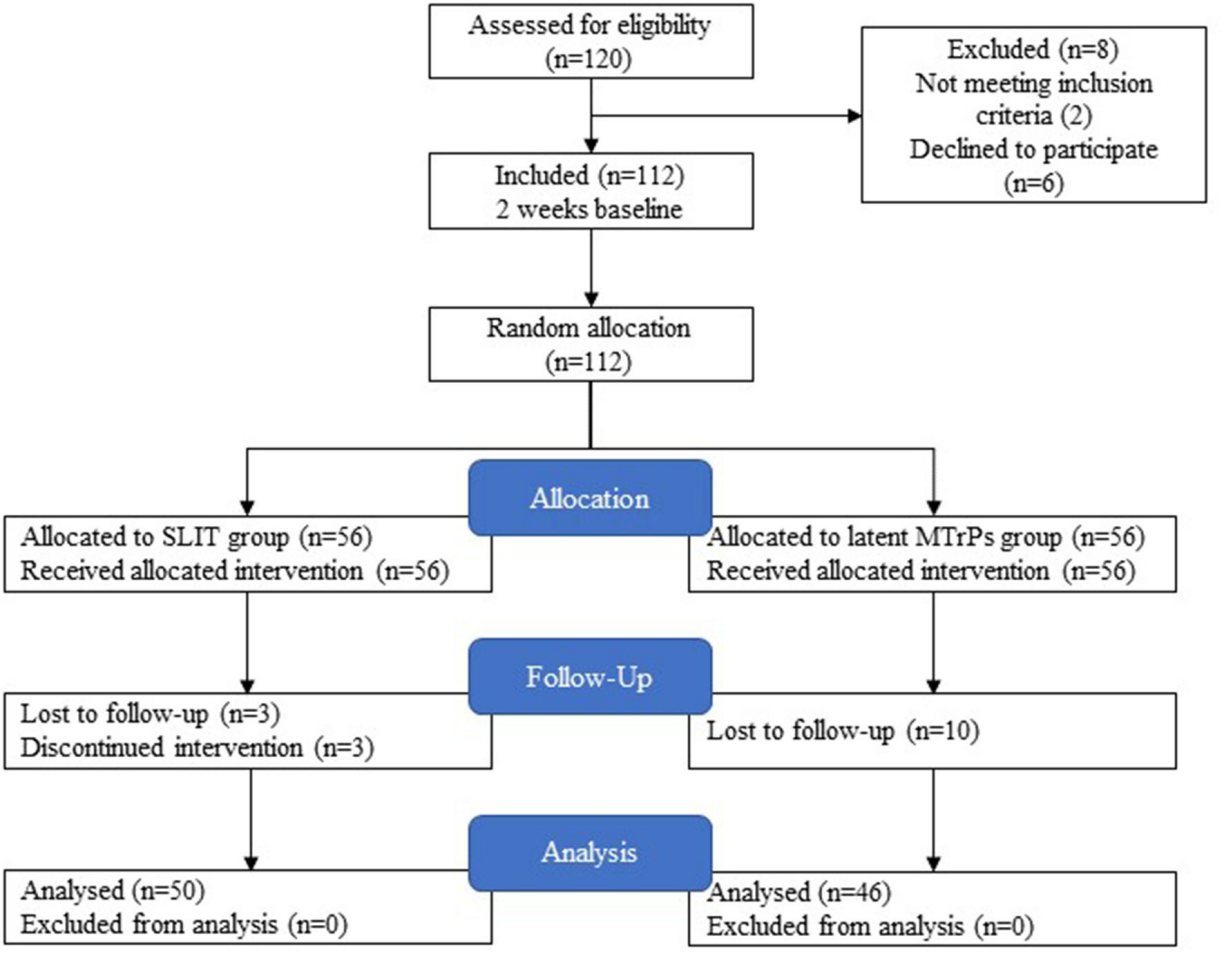
Flow diagram of trial procedure.

According to the recruitment strategy, 112 patients who had moderate to severe AR were enrolled in the trial. Thirteen patients were lost to follow-up, and three patients did not adhere to SLIT. Of them, 96 (85.7 %) completed the study. A total of 96 patients completed the trial. The participant flow diagram is shown in Figure 2. There was no significant difference between the two groups with regard to age, sex, duration of AR, or severity of symptoms of the patients before treatment. All demographics of the included participants were comparable (*p* > 0.05; Table 1).

**Table 1:** Demographic characteristics of included participants.

	**SLIT (*n* = 56)**	**Latent MTrPs(*n* = 56)**	**Significance**
Sex, *n* (%)			*χ^2^* = 0.322 *P =* 0.571
Male	27 (48)	30 (54)	
Female	29 (52)	26 (46)	
Age (y)			t = 1.034 *P* = 0.303
Mean (SD)	33 (11)	31 (15)	
Minimum-maximum	9–68	12–56	
Symptoms of allergic rhinitis, *n* (%)			
Persistent	56 (100)	56 (100)	
Severity of allergic rhinitis, *n* (%)			
Mild	0 (0)	0 (0)	
Moderate-severe	50 (100)	40 (100)	
Duration of allergic rhinitis, y (Mean ± SD)	6.18 ± 4.10	6.73 ± 3.51	t = 0.768 *P* = 0.444

### Efficacy

**Figure 3:**
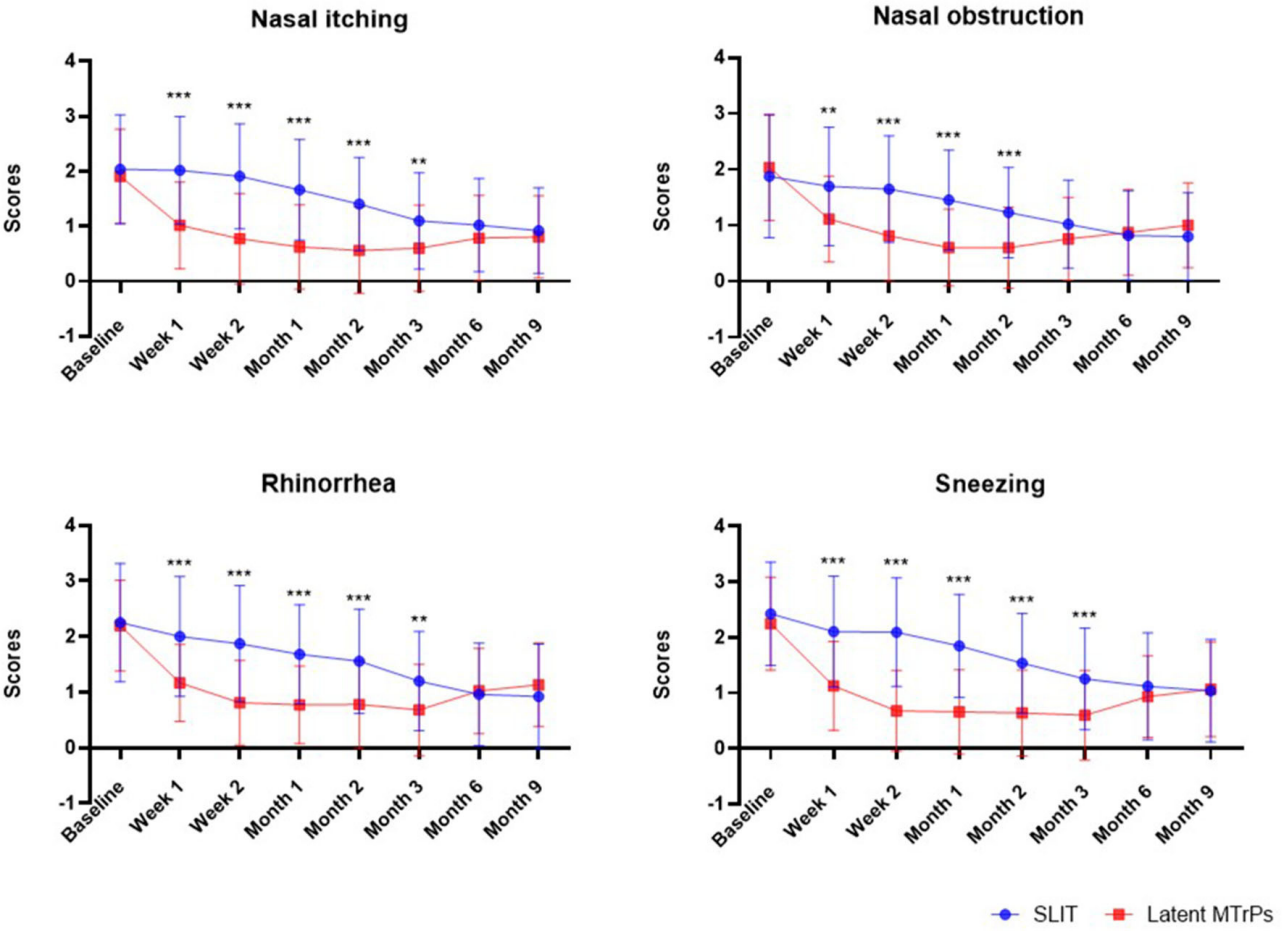
Effect of the SLIT and latent MTrPs injection on nasal and eye symptoms during the 9 months follow-up period in patient with AR. *: *p* < 0.05, **: *p* < 0.01, and ***: *p* < 0.001. SLIT, sublingual immunotherapy; MTrPs, myofascial trigger points.

**Figure 4:**
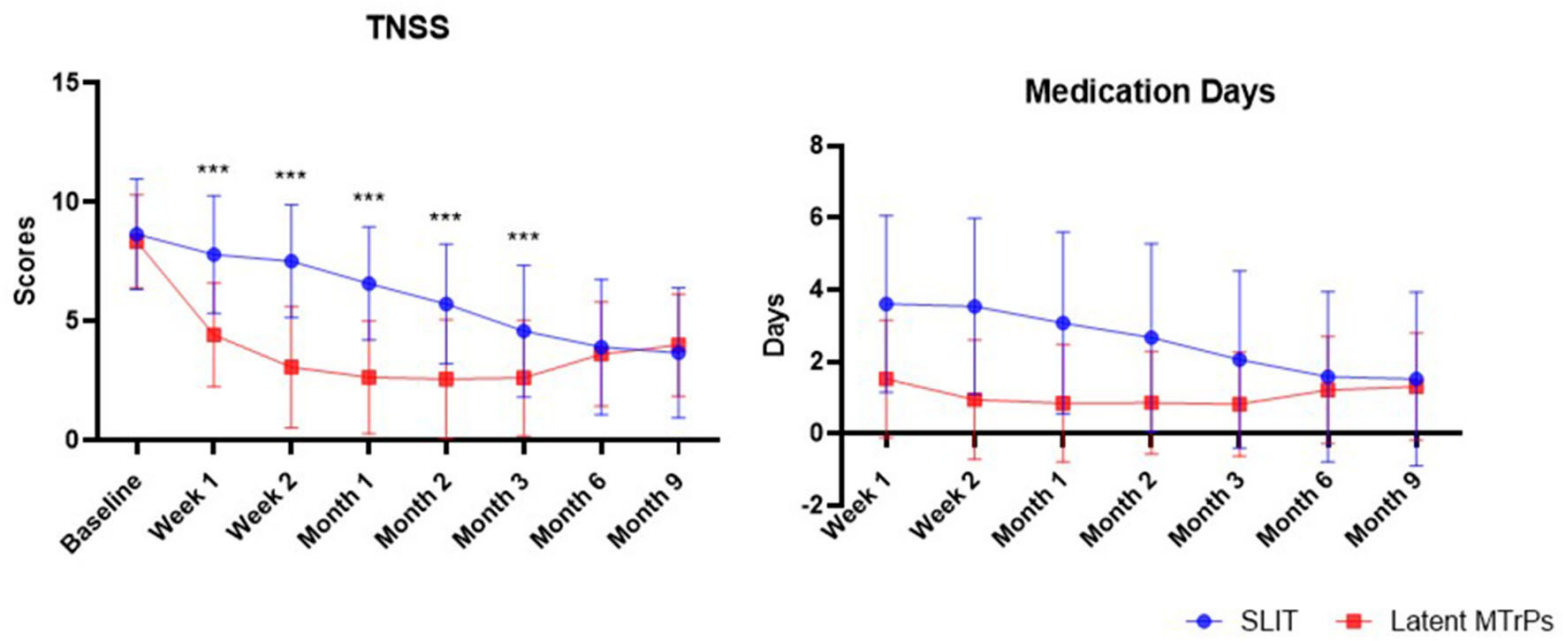
The trend of the changes of TNSS and number days of rescue medication usage during the past week prior to the assessment points. TNSS, total nasal symptom score.

After treatment, the latent MTrPs group showed a significantly reduction in TNSS and medications days compared to the SLIT group during 3 months after treatment. This improvement was maintained throughout the follow-up period, which indicates that the onset time of efficacy was at week 1. A statistically significant improvement in the TNSS was also observed in the SLIT group, which was maintained throughout the treatment period with the exception of week 1–8. The results for analyses of efficacy during the follow-up period are presented in Figures 3, 4 and Table 2. The TNSS at week 1, week 2, month 1, month 2, and month 3 for latent MTrPs and SLIT were 4.43 vs. 7.80, 3.08 vs. 7.52, 2.64 vs. 6.59, 2.58 vs. 5.73, and 2.62 vs. 4.59, respectively (mean difference; *p* < 0.001), which was statistically significant. At the month 6 and the end of the evaluation period (month 9), the differences between latent MTrPs and SLIT groups in mean TNSS and AR symptoms were not statistically significant (*p* = 0.947, *p* = 0.196, Table 2) but significantly reduced to TNSS a greater level from baseline (from 8.36 ± 1.96 to 4.00 ± 2.15, from 8.66 ± 2.31 to 3.68 ± 2.74). Moreover, the medication days for the latent MTrPs group were different from those for the SLIT group during the follow-up period (Figure 4). However, eight (17 %) patients reported that injection treatment did not work in the latent MTrPs group.

**Table 2:** Change from baseline in nasal symptoms and TNSS.

	**SLIT Mean (SD)**	**LMTrPs Mean (SD)**	***P***
**Baseline (*****n*** **=** **56;** ***n*** **=** **56)**			
Nasal itching	2.04 (0.99)	1.91 (0.86)	0.337
Nasal obstruction	1.88 (1.10)	2.04 (0.95)	0.506
Rhinorrhea	2.25 (1.07)	2.20 (0.82)	0.304
Sneezing	2.43 (0.93)	2.25 (0.84)	0.097
Total nasal symptom score	8.66 (2.31)	8.36 (1.96)	0.150
(TNSS)			
**Week 1 (*****n*** **=** **56;** ***n*** **=** **54)**
Nasal itching	2.02 (0.98)	1.02 (0.79)	<0.001
Nasal obstruction	1.70 (1.06)	1.11 (0.77)	0.003
Rhinorrhea	2.00 (1.08)	1.17 (0.69)	<0.001
Sneezing	2.11 (1.00)	1.13 (0.80)	<0.001
TNSS	7.80 (2.47)	4.43 (2.18)	<0.001
**Week 2 (*****n*** **=** **54;** ***n*** **=** **53)**
Nasal itching	1.91 (0.96)	0.77 (0.82)	<0.001
Nasal obstruction	1.65 (0.95)	0.81 (0.81)	<0.001
Rhinorrhea	1.87 (1.05)	0.81 (0.76)	<0.001
Sneezing	2.09 (0.98)	0.68 (0.73)	<0.001
TNSS	7.52 (2.37)	3.08 (2.55)	<0.001
**Month 1 (*****n*** **=** **53;** ***n*** **=** **53)**
Nasal itching	1.66 (0.92)	0.62 (0.77)	<0.001
Nasal obstruction	1.45 (0.89)	0.60 (0.69)	<0.001
Rhinorrhea	1.68 (0.89)	0.77 (0.70)	<0.001
Sneezing	1.85 (0.93)	0.66 (0.76)	<0.001
TNSS	6.59 (2.37)	2.64 (2.38)	<0.001
**Month 2 (*****n*** **=** **52;** ***n*** **=** **51)**
Nasal itching	1.40 (0.85)	0.56 (0.79)	<0.001
Nasal obstruction	1.23 (0.81)	0.60 (0.73)	<0.001
Rhinorrhea	1.56 (0.94)	0.78 (0.79)	<0.001
Sneezing	1.54 (0.90)	0.64 (0.78)	<0.001
TNSS	5.73 (2.51)	2.58 (2.49)	<0.001
**Month 3 (*****n*** **=** **51;** ***n*** **=** **51)**
Nasal itching	1.10 (0.88)	0.60 (0.78)	0.002
Nasal obstruction	1.02 (0.79)	0.76 (0.74)	0.104
Rhinorrhea	1.20 (0.89)	0.68 (0.82)	0.002
Sneezing	1.26 (0.91)	0.60 (0.81)	<0.001
TNSS	4.59 (2.77)	2.62 (2.43)	<0.001
**Month 6 (*****n*** **=** **50;** ***n*** **=** **48)**
Nasal itching	1.02 (0.84)	0.76 (0.77)	0.10
Nasal obstruction	0.82 (0.80)	0.87 (0.77)	0.735
Rhinorrhea	0.96 (0.92)	1.02 (0.77)	0.447
Sneezing	1.12 (0.96)	0.94 (0.73)	0.446
TNSS	3,92 (2.83)	3.62 (2.19)	0.947
**Month 9 (*****n*** **=** **50;** ***n*** **=** **46)**
Nasal itching	0.92 (0.78)	0.80 (0.75)	0.480
Nasal obstruction	0.80 (0.78)	1.00 (0.76)	0.206
Rhinorrhea	0.92 (0.94)	1.13 (0.75)	0.091
Sneezing	1.04 (0.92)	1.07 (0.85)	0.748
TNSS	3.68 (2.74)	4.00 (2.15)	0.196

### Onset Time of Efficacy

The onset time of efficacy in the SLIT group was longer than that in the latent MTrPs group. Twenty-two patients (39.3 %) felt relief immediately after the injection if they were experiencing AR symptoms, especially nasal obstruction, at that time. SLIT has a slower onset time, and the symptoms of most patients started to improve after approximately 2 months.

### Safety

There were some mild adverse events reported by participants during the treatment and follow-up period. Of those who reported to have noticed adverse events in the latent MTrPs group, seven (12.5 %) reported that they appeared to loss of appetite. Nausea (5, 8.9%) and headache (4, 7.1%) were the next most common events. In the SLIT group, throat irritation (18, 32.1%) was the most common, followed by loss of appetite (11, 19.6%), and next headache (6, 10.8%). Nine percent of patients (5/56, female) developed nausea after latent MTrPs injection, and this nausea was relieved within 30 minutes. Nausea may occasionally occur in some patients and can be alleviated by having the patient lie down on a bed for a while. Adverse events in the SLIT and latent MTrPs group are shown in Table 3.

**Table 3:** Treatment-related adverse events.

**Adverse events, *n* (%)**	**SLIT (*n* = 56)**	**Latent MTrPs (*n* = 56)**
Throat irritation	18 (32.1)	0 (0)
Loss of appetite	11 (19.6)	7 (12.5)
Headache	6 (10.8)	4 (7.1)
Nausea	1 (1.8)	5 (8.9)
Epistaxis	3 (5.4)	2 (3.6)
Common cold	4 (7.1)	3 (5.4)
Swollen tongue	2 (3.6)	0 (0)
Insomnia	2 (3.6)	3 (5.4)
Diarrhea	1 (1.8)	0 (0)
Significant weight gain	0 (0)	1 (1.8)
Facial acne	1 (1.8)	0 (0)

## Discussions

This study was the first trial to evaluate the efficacy and safety of latent MTrPs injection for AR. This study demonstrated that latent MTrPs injection could improve the symptoms of persistent, moderate to severe AR. Furthermore, the protection afforded by treatment with a single latent MTrPs injection session was sustained for several months. In this study, no serious adverse events attributable to latent MTrPs injection or SLIT occurred; five female patients reported nausea after latent MTrPs injection treatment, but it was alleviated quickly. We consider that the nausea was probably related to an imbalance caused by the dominance of the parasympathetic nerve (upper cervical vagus nerve) reflex caused by needle stimulation or by the nervous system of the patient.

In this study, another phenomenon was observed: the effect of latent MTrPs injection on AR was dependent on whether the patient could accurately feel the pain when the practitioner held the needle to perform latent MTrPs injection. This indicated that whether the drug could accurately intervene in latent MTrPs was critical. Only when the feedback of the patient is accurate can the drug be precisely injected near the latent MTrPs, otherwise, the effect may not be beneficial.

According to the theory of Simons, latent MTrPs can cause painful diseases and non-painful diseases and are often accompanied by autonomous nerve changes. In addition, many clinical and fundamental studies on AR and endogenous neuropeptides have been performed (18–23). Our previous research showed that a large number of inflammatory cytokines are expressed near latent MTrPs and that the abnormal contraction of one or more abnormal sarcomeres in taut bands forms latent MTrPs, as confirmed by morphological evidence (24, 25). As a result, we speculated that the relief of AR after latent MTrPs injection might be attributable to (1) the recovery of autonomic nerve network balance and (2) the relief of entrapped nerve inflammation. Latent MTrPs present in the head and neck muscles might induce neural inflammation when administered to the noses and eyes and affect the autonomic nerve network balance, causing AR symptoms. In this study, we showed the clinical efficacy of latent MTrPs injection for AR but did not elucidate the underlying molecular mechanism. Future studies are needed to confirm this interpretation.

### Relationship Between the Autonomic Nerve Network and Abnormal Mucous Gland Secretion in Patients With AR

Nasal innervation mainly involves the sensory nerve (trigeminal ganglion), sympathetic nerve (superior cervical ganglion), and parasympathetic nerve (sphenopalatine nerve). The nasal mucosal glands and nasal capillaries are innervated by the parasympathetic and sympathetic nerves. Neuropeptides, such as substance P (SP), calcitonin gene-related peptide (CGRP), and vasoactive intestinal peptide (VIP), secreted by the nervous system can cause vasodilation and alter vascular permeability while also activating glands and inflammatory cells (26). In addition, these cholinergic reflexes play a significant role in stimulating the abnormal secretion of mucous by the mucous glands (27). A prior study suggested that acupuncture at the sphenopalatine ganglion can improve nasal ventilation and affects the expression of neuropeptides (28). Vidian neurectomy is an option for refractory AR and has achieved great short-term and long-term results, but nearly half of patients develop dry eye (29). Similarly, transnasal resection of the posterior nasal nerve (TRPN) can improve the symptoms of patients with AR (30). Immune-neuronal disorders underlie the neuronal-based symptoms of allergies (31). These studies indicate that there is a close relationship between the autonomic nerve network and abnormal secretion by the mucous glands. Neural inflammation and an imbalance in the autonomic nerve network in the head and neck zones, especially the upper cervical sympathetic nerve and the parasympathetic nerve, are dominant mechanisms, resulting in the oversecretion of mucous by the nasopharyngeal mucous glands.

### Relationships Among Latent MTrPs, the Autonomic Nerve Network, and Abnormal Mucous Gland Secretion in Patients With AR

Poor or improper living habits, poor posture, lifting excessive weight, long-term hard physical labor, malnutrition, and sleep disorders are the major causes of myofascial tissue damage and latent MTrPs (8). These repeated insults cause abnormal contraction of the myofascial sarcomeres to form latent MTrPs and entrap the nerves. Latent MTrPs consist of one or more abnormally contracted sarcomeres and can result in the production of a large number of inflammatory cytokines that stimulate nearby or referred nerves. A variety of chronic factors can activate peripheral nerve terminals and promote the release of neuropeptides. Neuropeptides interact with immune cells, leading to the release of inflammatory factors, which results in inflammation. In addition, immune cells in peripheral nerve terminals and spinal cord release molecules that can regulate pain and, in turn, peripheral nerve endings and spinal cord can release neuropeptides to regulate immune cell responses (32). The interaction among latent MTrPs, inflammation, and the autonomic nerve network plays an important role in AR.

The effect of latent MTrPs on the autonomic nervous system might disrupt the balance between the sympathetic and parasympathetic nerves, leading to immune-neuronal disorder. In detail, various adverse factors contribute to myofascial tissue damage and form latent MTrPs accompanied by a large number of inflammatory cytokines. These inflammatory cytokines entrapped autonomic nerves that innervate the nose and eye. Latent MTrPs are likely to stimulate the autonomic nerve network in the head and neck zones *via* the cervical sympathetic nerve ganglion and the sphenopalatine ganglion, leading to abnormal secretion (hypersecretion or hyposecretion) of mucous by the mucous glands and inducing various AR symptoms. Inflammation of the referred entrapped nerves due to latent MTrPs and autonomic nerve network imbalance collectively result in immune-neuronal disorder.

We also evaluated the relationship between the persistence of the injected drugs and clinical duration in the latent MTrPs group. Among the drugs used, the maximum duration of drug persistence did not exceed 1 month. However, the relief of symptoms lasted for several months. Hence, we believe that the observed clinical improvements in AR were due to latent MTrPs rather than due to the effects of the injected drugs themselves. If the effects depended on only the duration of the drugs, the duration of AR symptom improvement should have been shorter than that observed. The latent MTrPs group has a faster onset time of efficacy. Twenty-two patients (39.3 %) felt the symptoms of nasal obstruction relieved immediately after latent MTrPs injection. The immediate relief of nasal obstruction may be related to the effects of lidocaine. Lidocaine can quickly exert a nerve block and reduce sympathetic nerve activity. In addition, lidocaine has been known to possess an anti-inflammatory effect.

### Relationship Between Latent MTrPs Injection and Chinese Acupuncture

A large number of studies have confirmed that acupuncture has a positive effect on AR (16, 33, 34). We consider that latent MTrPs are different from the acupoints used in traditional Chinese medicine. First, Chinese acupuncture considers fixed locations on the meridians, whereas latent MTrPs have different positions in skeletal muscles. Second, morphological or pathophysiological evidence of acupoints has not yet been confirmed, while latent MTrPs appear as one or more abnormally contracted sarcomeres under the microscope (25). Third, it usually takes 6–8 weeks to accomplish acupuncture procedures.

## Conclusions

Latent MTrPs injection provided long-term clinical efficacy with few adverse events in persistent, moderate to severe patients with AR. Latent MTrPs injection had a faster onset time and greater improvement effects than SLIT during a 3-month period. The potential underlying mechanisms might be that inflammation of the referred entrapped nerve because of latent MTrPs and autonomic nerve network imbalance results in abnormal secretion by the mucous glands. This study provided important evidence that latent MTrPs are associated with AR, future research will focus on the exact pathophysiological mechanisms of latent MTrPs and the function of autonomic nerve system imbalance with AR.

## Data Availability Statement

The raw data supporting the conclusions of this article will be made available by the authors, without undue reservation.

## Ethics Statement

The studies involving human participants were reviewed and approved by Human Research Ethics Committee of Qilu Hospital (KYLL-2017-611). Written informed consent to participate in this study was provided by the participants' legal guardian/next of kin. Written informed consent was obtained from the individual(s), and minor(s)' legal guardian/next of kin, for the publication of any potentially identifiable images or data included in this article.

## Author Contributions

YL performed the statistical analysis, drafted the manuscript. FQ designed the study and performed the latent MTrPs injection procedure, the discussion of study were incorporated into his Qi Points Medicine theory. YY participated in patients recruitment and guided the procedure of SLIT at the Department of Otolaryngology. HZ participated in the revision process of the manuscript and contributed her own ideas. QH and AB participated in patients recruitment and assessment the symptoms of AR. YH made a great contribution to the revision of manuscript. All authors contributed to the article and approved the submitted version.

## Funding

This study was funded by the National Natural Science Foundation of China (No. 81672250), the Fundamental Research Funding of Shandong University, the Qilu Hospital of Shandong University of China, and the Science and Technology Cooperation Center of Chinese-USA in Anesthesiology and Pain of Shandong Province.

## Conflict of Interest

The authors declare that the research was conducted in the absence of any commercial or financial relationships that could be construed as a potential conflict of interest.

## Publisher's Note

All claims expressed in this article are solely those of the authors and do not necessarily represent those of their affiliated organizations, or those of the publisher, the editors and the reviewers. Any product that may be evaluated in this article, or claim that may be made by its manufacturer, is not guaranteed or endorsed by the publisher.

## Abbreviations

AR: Allergic rhinitis
MTrPs: Myofascial trigger points
SLIT: Sublingual immunotherapy
TNSS: Total nasal symptom score
MPS: Myofascial pain syndrome
LTR: Local twitch response
SP: Substance P
CGRP: Calcitonin gene-related peptide
VIP: Vasoactive intestinal peptide
TRPN: Transnasal resection of the posterior nasal nerve.
